# Retracted: Efficacy of Crizotinib Combined with Chemotherapy in Treating Advanced Non-Small-Cell Lung Cancer and Effect on Patients' Quality of Life and Adverse Reaction Rate

**DOI:** 10.1155/2022/9834370

**Published:** 2022-11-23

**Authors:** Journal of Healthcare Engineering

*Journal of Healthcare Engineering* has retracted the article titled “Efficacy of Crizotinib Combined with Chemotherapy in Treating Advanced Non-Small-Cell Lung Cancer and Effect on Patients' Quality of Life and Adverse Reaction Rate” [1] due to concerns that the peer review process has been compromised.

Following an investigation conducted by the Hindawi Research Integrity team [2], significant concerns were identified with the peer reviewers assigned to this article; the investigation has concluded that the peer review process was compromised. We therefore can no longer trust the peer review process, and the article is being retracted with the agreement of the Chief Editor.
